# Supplementary figures and images for: Ptgds downregulation protect vestibular hair cells from aminoglycoside-induced vestibulotoxicity
Source: PLoS One. 2025 Apr 8;20(4):e0320634. doi: 10.1371/journal.pone.0320634 (PMC11978090; doi:10.1371/journal.pone.0320634)

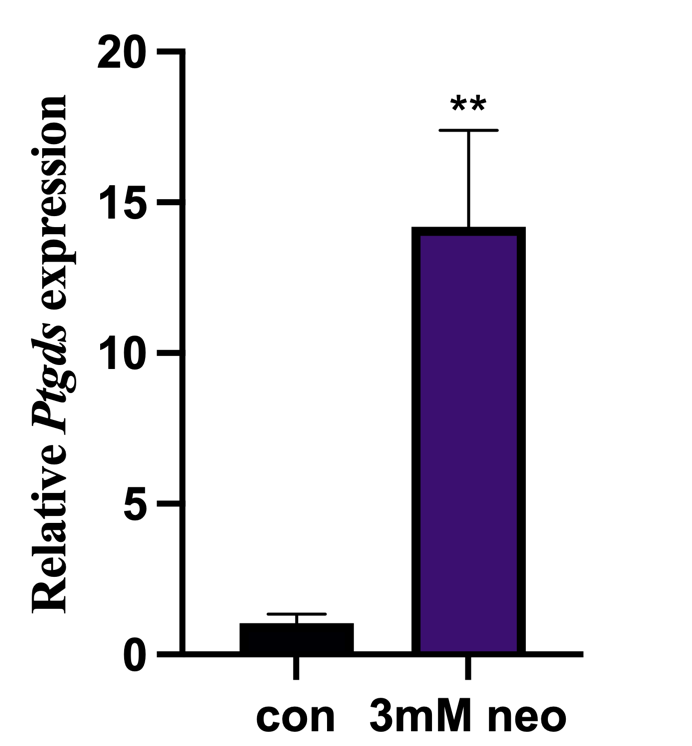

Supplement: S1 Fig — Validation of RNA results by qRT-PCR showing upregulation of Ptgds expression in the utricle following 3mM neomycin-induced injury. (TIFF) [file pone.0320634.s001.tiff]
