# Supplementary material for: Ptgds downregulation protect vestibular hair cells from aminoglycoside-induced vestibulotoxicity
Source: PLoS One. 2025 Apr 8;20(4):e0320634. doi: 10.1371/journal.pone.0320634 (PMC11978090; doi:10.1371/journal.pone.0320634)
Supplement: S1 Table — In Fig 1B, a highly significant treatment effect was observed (F = 265.6, P < 0.0001), indicating substantial differences between the treatment groups. Fig 1C also showed a significant effect (F = 1115, P < 0.0001), with distinct group differences. In Fig 2B, a large effect was found (F = 30.95, P < 0.0001), while Fig 2C and Fig 2D demonstrated moderate effects (F = 12.63, P = 0.0021 and F = 9.216, P = 0.0057, respectively). Fig 3B showed significant differences (F = 14.84, P = 0.0012), and Fig 3C-apaf1 had an exceptionally strong effect (F = 14260, P < 0.0001). For Fig 3C-cas3, the treatment effect was highly significant (F = 371.8, P < 0.0001), while Fig 3C-bcl2 revealed a moderate effect (F = 9.188, P = 0.0149). In Fig 4B, a strong treatment effect was observed (F = 52.65, P < 0.0001), and Fig 4C showed a very large treatment effect (F = 531.0, P < 0.0001). Finally, Fig 4D and Fig 4E exhibited significant treatment effects with moderate to large F values (F = 13.47, P = 0.0097 and F = 521.6, P < 0.0001, respectively). All the results demonstrate robust treatment effects with varying degrees of significance across the panels. (PDF) [file pone.0320634.s002.pdf]

Fig 1B

ANOVA table	SS	DF	MS	F (DFn, DFd)	P value
Treatment (between columns)	19123	5	3825	F (5, 24) = 265.6	P<0.0001
Residual (within columns)	345.6	24	14.40		
Total	19469	29			

Fig 1C

ANOVA table	SS	DF	MS	F (DFn, DFd)	P value
Treatment (between columns)	886.3	3	295.4	F (3, 8) = 1115	P<0.0001
Residual (within columns)	2.120	8	0.2651		
Total	888.4	11			

Fig 2B

ANOVA table	SS	DF	MS	F (DFn, DFd)	P value
Treatment (between columns)	9356	3	3119	F (3, 8) = 30.95	P<0.0001
Residual (within columns)	806.0	8	100.8		
Total	10162	11			

Fig 2C

ANOVA table	SS	DF	MS	F (DFn, DFd)	P value
Treatment (between columns)	12758	3	4253	F (3, 8) = 12.63	P=0.0021
Residual (within columns)	2695	8	336.8		
Total	15453	11			

Fig 2D

ANOVA table	SS	DF	MS	F (DFn, DFd)	P value
Treatment (between columns)	4855	3	1618	F (3, 8) = 9.216	P=0.0057
Residual (within columns)	1405	8	175.6		
Total	6260	11			

Fig 3B

ANOVA table	SS	DF	MS	F (DFn, DFd)	P value
Treatment (between columns)	107.6	3	35.86	F (3, 8) = 14.84	P=0.0012
Residual (within columns)	19.33	8	2.417		
Total	126.9	11			

Fig 3C-apaf1

ANOVA table	SS	DF	MS	F (DFn, DFd)	P value
Treatment (between columns)	13.59	2	6.797	F (2, 6) = 14260	P<0.0001
Residual (within columns)	0.002860	6	0.0004767		
Total	13.60	8			

Fig 3C-cas3

ANOVA table	SS	DF	MS	F (DFn, DFd)	P value
Treatment (between columns)	1.129	2	0.5645	F (2, 6) = 371.8	P<0.0001
Residual (within columns)	0.009111	6	0.001519		
Total	1.138	8			

Fig 3C-bcl2

ANOVA table	SS	DF	MS	F (DFn, DFd)	P value
Treatment (between columns)	4.507	2	2.254	F (2, 6) = 9.188	P=0.0149
Residual (within columns)	1.472	6	0.2453		
Total	5.979	8			

Fig 4B

ANOVA table	SS	DF	MS	F (DFn, DFd)	P value
Treatment (between columns)	2.061	3	0.6871	F (3, 8) = 52.65	P<0.0001
Residual (within columns)	0.1044	8	0.01305		
Total	2.166	11			

Fig 4C

ANOVA table	SS	DF	MS	F (DFn, DFd)	P value
Treatment (between columns)	15447	2	7724	F (2, 6) = 531.0	P<0.0001
Residual (within columns)	87.28	6	14.55		
Total	15534	8			

Fig 4D

ANOVA table	SS	DF	MS	F (DFn, DFd)	P value
Treatment (between columns)	2.295	2	1.147	F (2, 5) = 13.47	P=0.0097
Residual (within columns)	0.4258	5	0.08516		
Total	2.721	7			

Fig 4E

ANOVA table	SS	DF	MS	F (DFn, DFd)	P value
Treatment (between columns)	10995	2	5497	F (2, 6) = 521.6	P<0.0001
Residual (within columns)	63.23	6	10.54		
Total	11058	8			
