# Supplementary material for: Ptgds downregulation protect vestibular hair cells from aminoglycoside-induced vestibulotoxicity
Source: PLoS One. 2025 Apr 8;20(4):e0320634. doi: 10.1371/journal.pone.0320634 (PMC11978090; doi:10.1371/journal.pone.0320634)
Supplement: Attachment — Submitted filename: Chen_et_al_PLOS ONE_20240521_Shiyi.docx [file pone.0320634.s003.docx]

**Chen et al., Ptgds downregulation protect vestibular hair cells from aminoglycoside-induced vestibulotoxicity.**

**Summary:**

Chen and colleagues studied the expression and function of Ptgds in response to neomycin-induced injury. They showed that Ptgds is upregulated in neomycin-injured hair cells and suggested that such upregulation may contribute to the incidence of vertigo and other symptoms of vestibular dysfunction. The authors also found that knocking down Ptgds protects utricle explants and HEI-OC1 cells from neomycin-mediated injury by reducing the production of reactive oxygen species (ROS), the suppression of inflammation, and the abrogation of hair cell apoptosis.

This manuscript tried to highlight the role of Ptgds in aminoglycoside-induced vestibular dysfunction. However, my enthusiasm for this manuscript was significantly diminished by the lack of evidence demonstrating the authors’ proposed mechanism. I recommend this manuscript for publication after addressing the major and minor concerns below:

**Major concerns:**

1. **Lack of Protocol Validation and Reference support for in vitro neomycin treatment protocol:** In Fig. 1A, the authors claimed to have established an in vitro model of neonatal rodent utricle culture and treated cells with neomycin (3 mM) for 12 hours to induce neomycin-associated injury. However, the authors did not provide any detail on how this protocol was established and validated. Specifically, there is no mention of the rationale for the chosen neomycin concentration and treatment duration. Furthermore, the authors do not provide any references to support their protocol, making it difficult to assess the validity and reliability of the model. To strengthen the manuscript, please provide a more detailed description of the protocol, including references to established methods and validation experiments, to demonstrate the efficacy and specificity of the neomycin treatment.
2. **What makes the authors selectively focus on Ptgds?** In Fig. 1A, the authors have identified 782 significant differentially expressed genes (DEGs) in response to neomycin-associated injury, with 248 genes being downregulated. While the authors chose to focus on the gene Ptgds, it is unclear why this gene is prioritized over other significantly downregulated genes. The manuscript would benefit from a more comprehensive analysis and discussion of the downregulated genes, including their functional enrichment and potential roles in the context of neomycin-associated injury. A more inclusive approach would strengthen the manuscript and provide a more complete understanding of the gene expression changes occurring in response to neomycin-associated injury.
3. **No representative cell images for Fig. 1B:** The authors investigated the effects of various neomycin concentrations on HEI-OC1 cell survival, selecting a 30 mM concentration for future experiments based on a ~50% survival rate. However, the manuscript would greatly benefit from representative cell images to illustrate the effects of neomycin treatment on cell morphology and viability. Including images of control and treated cells would enhance the manuscript's clarity and facilitate a better understanding of the experimental outcomes.
4. **More evidence needed for the role of Ptgds in mitigating neomycin-induced oxidative stress:** In Fig. 4, the authors showed the role of Ptgds in mitigating neomycin-induced oxidative stress and apoptotic death in hair cells, but further exploration is needed to solidify the conclusions. To quantify ROS levels, a more precise method such as flow cytometry should be used to quantify the levels of ROS in each group, rather than relying solely on fluorescence signal intensity. In addition, it is important to examine ROS levels at various time points after neomycine treatment to understand the temporal dynamics of ROS accumulation and impact of Ptgds silencing. Furthermore, the authors should overexpress Ptdgs in HEI-OC1 cells to determine if this reverses the protective effects of Ptgds silecning on neomycin-induced ROS accumulation. The authors should also co-treat cells with antioxidants and neomycin to determine if reducing ROS levels can mimic the protective effects of Ptgds silencing.
5. **More evidence needed for the relationship between neomycin-induced damage and inflammatory activity in hair cells:** In Fig. 4, the authors present interesting findings on the relationship between neomycin-induced damage and inflammatory activity in hair cells, but further analysis is needed to support the claim. The protein levels of IL6, iNOS, and COX2 should be measured to confirm the changes observed at the mRNA level. It is also important to use immunofluorescence or immunohistochemistry to determine how Ptgds knockdown affects the expression patterns of IL6, iNOS, and COX2. In addition, the authors should examine the dose-dependent effects of neomycine on inflammatory activity and Ptgds expression to establish a clear correlation.
6. **Replace all bar graphs with superplots to enhance the clarity of the data:** The authors used bar graphs to present their data, which made it difficult to see individual data points and the variability within each group. Please replace bar graphs with superplots that clearly indicate individual data points and distinguish between individual experiments. This will provide a more accurate and transparent representation of the data, allowing readers to better understand the results and assess the significance of the findings. Please specify the statistical analysis being performed in every figure panel.

**Minor points:**

1. **Correct typos:** In Figures, cell viability was written as cell variability. Please correct.
2. **Add references:** In introduction, please cite the publications containing prior work for the sentence ‘While prior work conducted by our team has revealed the overexpression of Ptgds in vestibular inner ear hair cells in response to neomycin injury’
3. **Specify figures:** In result section 3.2, please add the corresponding figure after ‘as compared to those in which it had not (76.9% vs. 57.71%).’
