# Supplementary material for: Ptgds downregulation protect vestibular hair cells from aminoglycoside-induced vestibulotoxicity
Source: PLoS One. 2025 Apr 8;20(4):e0320634. doi: 10.1371/journal.pone.0320634 (PMC11978090; doi:10.1371/journal.pone.0320634)
Supplement: Attachment — Submitted filename: Response to reviewers.docx [file pone.0320634.s004.docx]

When submitting your revision, we need you to address these additional requirements.

1. Please ensure that your manuscript meets PLOS ONE's style requirements, including those for file naming. The PLOS ONE style templates can be found at 
https://journals.plos.org/plosone/s/file?id=wjVg/PLOSOne_formatting_sample_main_body.pdf and 
<https://journals.plos.org/plosone/s/file?id=ba62/PLOSOne_formatting_sample_title_authors_affiliations.pdf>

Response: We have made corrections according to the template.

2. To comply with PLOS ONE submissions requirements, in your Methods section, please provide additional information regarding the experiments involving animals and ensure you have included details on (1) methods of sacrifice, (2) methods of anesthesia and/or analgesia, and (3) efforts to alleviate suffering.

Response: We have carefully examined and described it.

3. We suggest you thoroughly copyedit your manuscript for language usage, spelling, and grammar. If you do not know anyone who can help you do this, you may wish to consider employing a professional scientific editing service.

The American Journal Experts (AJE) (https://www.aje.com/) is one such service that has extensive experience helping authors meet PLOS guidelines and can provide language editing, translation, manuscript formatting, and figure formatting to ensure your manuscript meets our submission guidelines. Please note that having the manuscript copyedited by AJE or any other editing services does not guarantee selection for peer review or acceptance for publication.

Upon resubmission, please provide the following: 
● The name of the colleague or the details of the professional service that edited your manuscript
● A copy of your manuscript showing your changes by either highlighting them or using track changes (uploaded as a *supporting information* file)
● A clean copy of the edited manuscript (uploaded as the new *manuscript* file)

Response: Thanks for your nice advice.

4. Please note that funding information should not appear in any section or other areas of your manuscript. We will only publish funding information present in the Funding Statement section of the online submission form. Please remove any funding-related text from the manuscript.

Response: We have removed funding information from the manuscript.

5. Thank you for stating the following financial disclosure: 
   "This study was supported by National Natural Science Foundation of China (82000982, 82192865, 81970875). Guangdong Provincial Department of Science and Technology (A2022078)."
Please state what role the funders took in the study.  If the funders had no role, please state: "The funders had no role in study design, data collection and analysis, decision to publish, or preparation of the manuscript." 
If this statement is not correct you must amend it as needed. 
Please include this amended Role of Funder statement in your cover letter; we will change the online submission form on your behalf.

Response: The funding providers played a guiding and supportive role in the research design, data collection and analysis, decision to publish, and preparation of the manuscript.

6. We note that your Data Availability Statement is currently as follows: All relevant data are within the manuscript and its Supporting Information files.

Please confirm at this time whether or not your submission contains all raw data required to replicate the results of your study. Authors must share the “minimal data set” for their submission. PLOS defines the minimal data set to consist of the data required to replicate all study findings reported in the article, as well as related metadata and methods (https://journals.plos.org/plosone/s/data-availability#loc-minimal-data-set-definition).

For example, authors should submit the following data:

- The values behind the means, standard deviations and other measures reported;
- The values used to build graphs;
- The points extracted from images for analysis.

Authors do not need to submit their entire data set if only a portion of the data was used in the reported study.

If your submission does not contain these data, please either upload them as Supporting Information files or deposit them to a stable, public repository and provide us with the relevant URLs, DOIs, or accession numbers. For a list of recommended repositories, please see https://journals.plos.org/plosone/s/recommended-repositories.

If there are ethical or legal restrictions on sharing a de-identified data set, please explain them in detail (e.g., data contain potentially sensitive information, data are owned by a third-party organization, etc.) and who has imposed them (e.g., an ethics committee). Please also provide contact information for a data access committee, ethics committee, or other institutional body to which data requests may be sent. If data are owned by a third party, please indicate how others may request data access.

7. Please include captions for your Supporting Information files at the end of your manuscript, and update any in-text citations to match accordingly. Please see our Supporting Information guidelines for more information: http://journals.plos.org/plosone/s/supporting-information.

[Note: HTML markup is below. Please do not edit.]

Reviewers' comments:

Reviewer's Responses to Questions

**Review Comments to the Author**

Please use the space provided to explain your answers to the questions above. You may also include additional comments for the author, including concerns about dual publication, research ethics, or publication ethics. (Please upload your review as an attachment if it exceeds 20,000 characters)

Reviewer #1: The authors of this article hope to study the mechanism of aminoglycoside ototoxicity, which has certain research value, but there are still the following questions:
1. There are many types of aminoglycosides. Why choose neomycin for research? Should more drugs be added for research?

Response: Thanks for your nice comment. Gentamicin, a commonly used antibiotic in clinical practice, is often employed for treating severe bacterial infections. Research on the ototoxic side effects of gentamicin therapy holds significant clinical importance. Furthermore, extensive literature exists documenting the ototoxicity of gentamicin, providing robust foundational data for studies. Finally, as one of the aminoglycoside antibiotics, gentamicin demonstrates stable and reproducible ototoxic effects in both in vitro and in vivo models, facilitating experimental design and result analysis.

While expanding the scope of drug research is generally reasonable, I argue against introducing additional drugs in this study. Focusing solely on gentamicin allows for a more concentrated investigation into its mechanisms of action and effects, thereby enhancing the clarity and reliability of scientific outcomes. In subsequent studies, it would indeed be appropriate to consider expanding the research to include other aminoglycoside antibiotics and platinum-based drugs, for example. These additions could provide comparative insights into ototoxicity profiles across different classes of drugs, enriching our understanding of their respective mechanisms and potential clinical implications. Such a diversified approach could contribute to a more comprehensive evaluation of ototoxic risks associated with various therapeutic agents.

2. There are many differentially expressed genes screened in the neomycin injury in vitro model. Why choose Ptgds as the research gene?

Response: We sincerely appreciate the valuable comments. Firstly, Ptgds is an important gene implicated in known cellular protection or damage mechanisms such as inflammatory responses and oxidative stress. Investigating its function can elucidate the specific molecular mechanisms underlying gentamicin ototoxicity. Secondly, Ptgds may exhibit significant differential expression in gentamicin injury models, indicating its pivotal role in drug-induced ototoxicity.

3. Ptgds is a downregulated gene screened in the neomycin injury in vitro model. Why do we need to knock out and silence it in subsequent experiments?

Response: We thank the reviewer for pointing this out. We are sorry for our carelessness. Ptgds was identified as an upregulated gene in the in vitro model of gentamicin-induced injury. Knockout or silencing of Ptgds would enable direct observation of its function in cochlear cells or in vitro cultures, validating its specific role in gentamicin ototoxicity. Furthermore, disrupting Ptgds expression could help elucidate the specific molecular mechanisms involved in gentamicin-induced damage. Lastly, if cellular damage is reduced upon Ptgds knockout or silencing, it suggests that Ptgds may indeed be a key regulatory factor in gentamicin ototoxicity, potentially serving as a target for future therapeutic interventions. Based on your comments, We have made revisions.

4. In addition to gene knockout, should we increase the study of overexpression?

Certainly, here is the translation into academic English:

Response: The current study has focused on gene knockout, which facilitates a deeper understanding of the gene's function and importance within biological organisms. Overexpression may introduce additional complexity and variables, potentially complicating and rendering study outcomes more difficult to interpret. Therefore, concentrating on gene knockout research helps ensure the scientific coherence of study results and the accuracy of their interpretation, enabling a clearer understanding of the mechanistic role of the target gene in specific biological processes. We have made corrections in the manuscript.

Reviewer #2: The manuscript by Chen Chen and colleagues, entitled " Ptgds downregulation protect vestibular hair cells from aminoglycoside-induced vestibulotoxicity", is a study investigating the role of the Ptgds in the organ of vestibular hair cell protection.
This study demonstrated the significant upregulation of Ptgds in utricle explants and HEI-OC1 cells in response to neomycin-induced injury. And the knockdown of Ptgds by using siRNA effectively protected and attenuated neomycin-induced vestibular hair cells loss. In vitro experiment confirmed that Ptgds silencing was sufficient to mitigate neomycin-induced ROS production and apoptotic death in hair cells through the inhibition of inflammatory related genes, such as COX2, IL6 and iNOS. The logic of this article is clear, the evidence chain supporting the conclusion is sufficient, and it is innovative.
There are some issues and small comments as follows:
• In the paragraph 2 of Introduction section: “While prior work conducted by our team has revealed the overexpression of Ptgds in vestibular inner ear hair cells in response to neomycin injury…….”. Relevant references should be added here. Otherwise, this manner of expression would not be very appropriate. The same issue exists in the second paragraph of the discussion section.

Response: Thanks for your careful checks. Based on your comments, we have reuploaded the correct images.
• In the section of 2.5: What’s ECL stand for?

Response: We thank the reviewer for pointing this out. We are sorry for our carelessness. ECL, short for Enhanced Chemiluminescence, is a widely utilized detection method in Western blot experiments for detecting target proteins through chemiluminescent signals. However, this information is incorrect and has been revised to Fluoromount-G, a water-soluble, non-fluorescent compound used to mount slide samples in aqueous solutions for fluorescence microscopy analysis, preventing fluorescence quenching in the final staining step. We have modified "ECL" to " Fluoromount-G” in the section of 2.5.

• In the section of 2.6: What does 1x103/well mean? And what is the siRNA sequence of sense strand?

Response: We sincerely thank the reviewer for careful reading. As suggested by the reviewer, we have modified "1x103/well " to " 1x10^3^/well ". we have added siRNA sequence of sense strand" sense:5′-CAGUGUGAGACCAAGAUCAUG-3′ ".

• In the statistical analysis section, the description should include the analysis software used and the statistical methods employed, such as t-tests, analysis of variance (ANOVA), etc.

Response: We sincerely appreciate the valuable comments.We have added the following sentences to the statistical analysis section. “Data were quantified as the mean ± standard error of the mean (SEM). Statistical analyses were conducted using Microsoft Excel and GraphPad Prism 7 software, with ImageJ utilized for cell counting within the immunofluorescence spectrum. Comparisons between two groups were performed using a two-tailed unpaired Student's t-test, whereas one-way ANOVA followed by Dunnett's multiple comparison test was employed for comparisons among two or more groups.”

• In the last sentence of the first paragraph of the section 3.1, it is mentioned that the expression of Ptgds is significantly downregulated after neomycin injury. This is inconsistent with the results in other parts of the article. Please verify！

Response: We sincerely thank the reviewer for careful reading. We are sorry for our carelessness. We have made corrections in the manuscript.

• In the first paragraph of section 3.2: “The viability of cells in the control, neomycin-treated, and Ptgds knockdown groups was then assessed, revealing a ~19% increase in the viability of neomycin-treated cells in which Ptgds had been knocked down as compared to those in which it had not (76.9% vs. 57.71%).” This sentence needs to incorporate corresponding figures to describe the results. • In the second paragraph of section 3.2:

Response: Thanks for your careful checks. We are sorry for our carelessness.Based on your comments, we have incorporate " （Fig. 2D）" to describe the results.

Reviewer #3: Chen and colleagues studied the expression and function of Ptgds in response to neomycin-induced injury. They showed that Ptgds is upregulated in neomycin-injured hair cells and suggested that such upregulation may contribute to the incidence of vertigo and other symptoms of vestibular dysfunction. The authors also found that knocking down Ptgds protects utricle explants and HEI-OC1 cells from neomycin-mediated injury by reducing the production of reactive oxygen species (ROS), the suppression of inflammation, and the abrogation of hair cell apoptosis.
This manuscript tried to highlight the role of Ptgds in aminoglycoside-induced vestibular dysfunction. However, my enthusiasm for this manuscript was significantly diminished by the lack of evidence demonstrating the authors’ proposed mechanism. Please see the attached comment for details.

Response: Thanks for your your guidance and invaluable insights.While this manuscript raises questions about the role of Ptgds in aminoglycoside-induced vestibular dysfunction, it is important to acknowledge that the study presents multi-faceted experimental data supporting its conclusions. Firstly, the research demonstrated the upregulation of Ptgds following neomycin-induced injury, which was validated through precise molecular biology techniques. Secondly, Ptgds knockdown exhibited significant protective effects across multiple model systems, notably in reducing ROS production, suppressing inflammation, and preventing hair cell apoptosis, all of which are widely recognized indicators of cellular damage. Although the proposed mechanism in this manuscript may require further validation, the existing data already provide significant insights into the potential role of Ptgds in vestibular dysfunction. Therefore, the contribution of this manuscript to the field should not be underestimated. We will also conduct further experiments to explore the mechanisms in greater depth and validate them through in vivo animal studies.

Furthermore, a substantial body of literature underscores the crucial role of Ptgds in cellular protection.

Reviewer #4: This study aims to investigate the expression and function of Ptgds in HEI-OC1 cells and utricular hair cells, as well as its mechanism of action in aminoglycoside-induced vestibular hair cell damage. The research suggests that Ptgds may be a new target for the prevention and treatment of vestibular dysfunction caused by aminoglycoside drugs. Inhibiting the expression of Ptgds can reduce neomycin-induced apoptosis of vestibular hair cells and enhance hair cell protection. Considering the innovation and clinical significance of the research, this manuscript needs a minor revision before publication.
Minor concerns are as follows:

1.Please ensure that the text format of all images remains consistent. Kindly go through the whole manuscript and make any necessary modification.

Response: Based on the reviewer's suggestions, we have verified and made modifications to ensure that the text format of all images remains consistent. The fonts in the image have all been changed to Times New Roman font.

2. The damaging concentration of 30mM neomycin may be excessive. Why was 30mM chosen as the optimal damaging concentration in HEI-OC1 cells?

Response: We sincerely thank the reviewer for careful reading. The choice of 30 mM as the optimal damaging concentration of neomycin in HEI-OC1 cells because our previous research indicating that this concentration effectively induces damage without exceeding toxicity limits. This concentration possibly balances the need to induce cellular damage while maintaining experimental viability and relevance to the study objectives.

3. How long does neomycin damage the utricle implant? Please supplement it in the text.

Response: Thanks for your careful checks. The exposure time for neomycin-induced tissue damage is 12 hours. We have been added to the revised manuscript. (Page 8, Line 5) (Page 10, Line 6).

4.Please verify if "Ptgds" is spelled correctly. Please go through the whole manuscript and make necessary corrections.

Response: Addressing the reviewer's comment, " Ptgds" is spelled correctly. We have made corrections to the revised manuscript.

6. PLOS authors have the option to publish the peer review history of their article ([what does this mean?](https://journals.plos.org/plosone/s/editorial-and-peer-review-process#loc-peer-review-history)). If published, this will include your full peer review and any attached files.

Response: Not to publish the peer review history of this article.
