# Supplementary material for: Ptgds downregulation protect vestibular hair cells from aminoglycoside-induced vestibulotoxicity
Source: PLoS One. 2025 Apr 8;20(4):e0320634. doi: 10.1371/journal.pone.0320634 (PMC11978090; doi:10.1371/journal.pone.0320634)
Supplement: Attachment — Submitted filename: Response to Reviewer#1.docx [file pone.0320634.s005.docx]

Reviewer #1: I don't agree with your response for comment 1. Since ototoxic antibiotics could be replaced by many other antibiotics without ototoxic, is this research meaningful? The author should clarify this.

Response: Thanks for your nice comment.

Ototoxic antibiotics, primarily referring to aminoglycosides, are irreplaceable in certain clinical scenarios where their therapeutic efficacy cannot be matched by other antibiotics without incurring ototoxic side effects. In specific circumstances, such as:

1) Treatment of resistant bacterial infections: Neomycin exhibits particular efficacy against resistant strains, such as multidrug-resistant Pseudomonas aeruginosa. Neomycin may be the only viable therapeutic option when common antibiotics fail due to resistance.

2) Specific clinical conditions: In cases where antibiotic options are constrained, such as in patients with drug allergies or contraindications to other medications, neomycin may be considered as an alternative treatment. Furthermore, advancing our understanding of the mechanisms underlying ototoxicity in these antibiotics can facilitate the development of strategies to mitigate adverse effects, such as dosage adjustments or combination therapies that reduce toxicity. This research also supports the design and development of new antibiotics that minimize ototoxicity while preserving or enhancing therapeutic efficacy. Notably, the ototoxicity of drugs is not limited to aminoglycosides; chemotherapeutic agents like cisplatin also exhibit ototoxicity. An in-depth investigation of these ototoxic drugs holds significant clinical importance for optimizing their use, reducing adverse effects, and driving the development and refinement of safer therapeutic options.
