# Supplementary material for: Ptgds downregulation protect vestibular hair cells from aminoglycoside-induced vestibulotoxicity
Source: PLoS One. 2025 Apr 8;20(4):e0320634. doi: 10.1371/journal.pone.0320634 (PMC11978090; doi:10.1371/journal.pone.0320634)
Supplement: Attachment — Submitted filename: response_to_reviewers_auresp_3.docx [file pone.0320634.s006.docx]

Dear Reviewer,

We sincerely appreciate your valuable comments and constructive feedback on our manuscript. Your suggestions have been instrumental in improving the quality of our study. Below, we provide detailed responses and revisions to address the issues you raised:

**Regarding the Legend of Figure 1C**

Thank you for pointing out the lack of clarity in the figure legend regarding the experimental group descriptions. We have revised the legend for Figure 1C and included detailed explanations of each experimental group in the revised manuscript to ensure clarity and comprehensibility. We hope this modification makes this section more intuitive and accurate.

**Regarding the Rigor of the Conclusion on Ptgds Upregulation**

We completely agree We fully agree with your suggestion that further validation of RNA experimental results is critical for enhancing the rigor of our conclusions. In fact, we have already conducted qRT-PCR experiments to validate these results, and the corresponding data have been incorporated into the revised manuscript (Supplementary Figure 1). The data clearly demonstrate the significant upregulation of Ptgds expression in the utricle following neomycin-induced damage. Based on these findings, we have also revised the discussion section to ensure a more scientifically robust conclusion.

**Regarding the Quality of Figure 3A**

Thank you for highlighting the quality issues with Figure 3A. Upon careful reevaluation, we found that due to technical limitations, there are spatial discrepancies between the TUNEL and DAPI signals, which prevent complete overlap. Specifically, DAPI, as a nuclear marker, exhibits uniform distribution, whereas Myosin7a selectively marks the stereocilia and cytoplasmic regions of cochlear and vestibular hair cells, reflecting its high spatial specificity. Following neomycin-induced damage, partial injury or death of vestibular hair cells resulted in Myosin7a signals covering only a subset of the DAPI regions. These findings are technically reasonable and align with the functional and biological differences between these two markers.

**Regarding the Improvement of English Writing**

Thank you for pointing out areas in need of improvement in the English writing. We conducted a comprehensive review of the manuscript and engaged professional editing services to optimize the language. These efforts were aimed at ensuring clarity and professionalism in presenting our findings. We believe these improvements have significantly enhanced the overall quality of the manuscript.

We sincerely thank you for your valuable suggestions, which have greatly contributed to refining our work. We hope these revisions adequately address your concerns. Should you have any further questions or recommendations, we are happy to make additional adjustments.

Once again, thank you for your support and constructive feedback.
