# Supplementary material for: Ptgds downregulation protect vestibular hair cells from aminoglycoside-induced vestibulotoxicity
Source: PLoS One. 2025 Apr 8;20(4):e0320634. doi: 10.1371/journal.pone.0320634 (PMC11978090; doi:10.1371/journal.pone.0320634)
Supplement: Attachment — Submitted filename: Response_to_Reviewers_auresp_4.docx [file pone.0320634.s007.docx]

We sincerely appreciate your valuable comments and constructive feedback on our manuscript. Your suggestions have been instrumental in improving the quality of our study. Below, we provide detailed responses and revisions to address the issues you raised:

1. All typographical errors in Table 1 have been corrected. In accordance with the nomenclature conventions outlined in the PLOS ONE submission guidelines, non-human gene names have been italicized, while human gene names are presented in uppercase.
2. The Materials and Methods section has been expanded to include the following details: “*After immunofluorescence staining, images were captured using a Zeiss LSM 800 confocal scanning microscope. Excitation light at two different wavelengths, 488 nm and 750 nm, was employed. The image resolution was set to 1024×1024 pixels, utilizing 20× and 63× magnifications, and the scanning speed was adjusted to 8 (with modifications made as needed based on image quality). The utricle was divided into the striolar region and the extrastriolar region. In each utricle, random 100 μm × 100 μm areas were selected from both the striolar and extrastriolar regions for imaging. The number of myosin7a-positive cells was manually counted using ImageJ software (NIH, USA). CellROX-stained images were acquired at a resolution of 1024×1024 pixels under 20× magnification using a REVOLVE FL microscope (Discover Echo, USA).*”
3. The data related to the numerator, denominator, and F-values for all ANOVA analyses (Figure 1B, C; Figure 2B, C, D; Figure 3B; Figure 4B, C, D, and E) have been transferred to the supplementary data section.
4. We are grateful for your insightful comments. The corresponding clarifications have been integrated into the manuscript:“*Compared to the negative control group, the expression of Ptgds in HEI-OC1 cells was significantly suppressed by approximately 70% after 48 hours of siRNA transfection.*”
5. A comprehensive description of the CCK-8 assay methodology has been incorporated into the Materials and Methods section: “*The CCK-8 (Cell Counting Kit-8) assay is utilized to evaluate cell viability or apoptosis. Initially, 1.2 × 10⁴ cells per well were seeded into a 96-well plate and allowed to adhere for 12 hours. Experimental groups were then established, encompassing a neomycin concentration gradient (0–50 mM, with a minimum of three replicates per group), a control group, a neomycin-treated group, and a neomycin-treated + siRNA group. Following 48 hours of transfection, neomycin was administered. After 24 hours of neomycin exposure, a 10:1 mixture of DMEM and CCK-8 solution was prepared, with 100 μL added to each well, followed by incubation at 37°C in the dark for 2 hours. Finally, absorbance at 450 nm was measured using a microplate reader, and cell viability (%) was calculated as (ODexperimental well – ODblank well) / (ODcontrol well – ODblank well) × 100%.*”
6. A description of the HEI-OC1 cell line has been added to the Materials and Methods section: *"HEI-OC1 cells are an immortalized mouse cochlear cell line widely utilized in research on ototoxicity and auditory-related mechanisms."*
7. Due to the significantly lower number of hair cells in the utricle compared to HEI-OC1 cells cultured in 96-well plates, it was necessary to proportionally reduce the neomycin concentration and exposure time for neonatal mouse utricle damage. Based on a review of the relevant literature and experimental findings from our team, 3 mM neomycin exposure for 12 hours was determined to be the optimal concentration and duration for inducing approximately 50% utricle damage. The reduced neomycin concentration (3 mM) employed in the TUNEL assay was specifically chosen to induce apoptosis without causing excessive cell death, thereby facilitating clearer detection of apoptotic cells.
8. We appreciate your comment regarding the statistical analysis. We would like to clarify that only one independent variable (treatment) was involved in this analysis. Although the data from the three genes were presented together in a single graph for visualization purposes, each gene was analyzed separately using one-way ANOVA. Therefore, a two-way ANOVA is not applicable in this context. The detailed statistical results have been provided in the supplementary data file.
9. The Discussion section has been expanded to elucidate the correlation between the neomycin concentrations used in this study and those encountered in clinical practice: “*Aminoglycosides (AGs) are a class of antibiotics widely used in clinical practice, known not only for their ototoxicity but also for their vestibulotoxicity. During clinical administration, AGs frequently cause damage to vestibular hair cells, which can lead to vestibular dysfunction. This dysfunction manifests as clinical symptoms such as ataxia, postural instability, dizziness, and vomiting. Neomycin, one of the commonly used aminoglycosides, is typically administered topically or orally in clinical settings. These routes of administration result in relatively low plasma concentrations of neomycin, thereby minimizing systemic exposure. However, despite the lower systemic absorption, neomycin is still associated with significant nephrotoxicity and ototoxicity. Due to these adverse effects, intravenous administration of neomycin is rarely employed in clinical practice* [*2*]*.*

*While neomycin demonstrates favorable therapeutic efficacy, its clinical application is severely limited by its unavoidable side effects, particularly its toxicity to the auditory and vestibular systems. In this study, the concentration of neomycin used exceeds the typical plasma concentrations observed during clinical use. However, it aligns with concentrations commonly employed in in vitro studies designed to model ototoxicity. This approach enables a more accurate investigation of the mechanisms underlying aminoglycoside-induced vestibular damage, offering valuable insights that may contribute to the development of strategies aimed at mitigating these adverse effects in clinical practice.*”
